# Beyond Hematology—Current Insights into Chimeric Antigen Receptor (CAR) T-Cell Therapy for Skin and Connective Tissue Disorders

**DOI:** 10.3390/cells15100874

**Published:** 2026-05-12

**Authors:** Agata Ciosek, Julia Hofmann, Kacper Galant, M. Peter Marinkovich, Agnieszka Wierzbowska, Magdalena Ciążyńska, Natalia Bień, Joanna Narbutt, Aleksandra Lesiak

**Affiliations:** 1 Student Scientific Research Club of Experimental, Clinical and Procedural Dermatology, Department of Dermatology, Pediatric Dermatology and Oncology, Medical University of Lodz, 90-419 Lodz, Poland; julia.kolodziejska@student.umed.lodz.pl; 2 Faculty of Medicine, Medical University of Lodz, 90-419 Lodz, Poland; kacpergalant.ld@gmail.com; 3 Program in Epithelial Biology, Department of Dermatology, Stanford University School of Medicine, Stanford, CA 94063, USA; 4 Department of Dermatology, Palo Alto Veterans Affairs Medical Center, Palo Alto, CA 94304, USA; 5 Department of Hematology, Medical University of Lodz, 93-513 Lodz, Poland; 6 Department of Dermatology, Pediatric Dermatology and Dermatological Oncology, Medical University of Lodz, 92-213 Lodz, Poland; 7 Laboratory of Autoinflammatory-Genetic and Rare Skin Disorders, Department of Dermatology, Pediatric Dermatology and Dermatological Oncology, Medical University of Lodz, 90-419 Lodz, Poland

**Keywords:** CAR T-cell therapy, chimeric antigen receptor T cells, adoptive cell therapy, immunotherapy, melanoma, autoimmune diseases, systemic lupus erythematosus, cutaneous lymphoma, systemic sclerosis, tumor microenvironment, cytokine release syndrome, CD19

## Abstract

**Highlights:**

**What are the main findings?**

- CD19 CAR T-cell therapy provides immune reset with reduced levels of autoreactive antibodies and clinical improvement in patients with SLE and SSc;
- Melanoma trials demonstrate CAR T expansion and activity but limited tumor infiltration and clinical efficacy;
- CCR4.CD30 CAR T therapy leads to tumor reduction or disease stabilization in early CTCL studies;

**What are the implications of the main findings?**

- The results displayed limited durable immune reset with persistent plasma cells, tumor and disease heterogeneity, antigen overlap/loss, infiltration barriers, resistance mechanisms, and T-cell depletion, collectively limiting response durability and safety;
- The main adverse events include grade 1–2 cytokine release syndrome; neurotoxic events are rare.

**Abstract:**

Chimeric antigen receptor (CAR) T-cell therapy represents a major advance in modern immunotherapy. This narrative review summarizes evidence from the past five years, including case reports, case series, and clinical trials, on its application beyond hematologic malignancies, focusing on autoimmune diseases such as systemic lupus erythematosus (SLE), systemic sclerosis (SSc), as well as solid tumors including melanoma and primary cutaneous lymphomas. CD19-directed CAR T-cells have demonstrated clinical benefits in SLE and SSc, with sustained immune reset, reduced autoreactive antibody levels, and clinical improvement. In melanoma, CAR T-cells targeting GD2, cMET, and CD20 have shown in vivo expansion and tumor infiltration; however, clinical efficacy remains limited, with transient stabilization or disease progression in most patients. In primary cutaneous lymphomas, early-phase studies with anti-CD70 and anti-CCR4.30 CAR T-cells indicate partial tumor regression and disease stabilization, often requiring additional therapy. Key challenges include limited durability of immune reset due to persistent plasma cells in autoimmune disorders, tumor heterogeneity, antigen loss or overlap, infiltration barriers, resistance mechanisms, and T-cell depletion in solid tumors, collectively reducing response durability and safety. The main toxicities include grade 1–2 cytokine release syndrome and rare hematologic complications, while immune effector cell-associated neurotoxicity syndrome is uncommon. Clinical translation remains limited and requires larger studies to improve efficacy and define safety profiles.

## 1. Introduction

### 1.1. Structural Characteristics of Chimeric Antigen Receptors

Initially developed to treat hematological malignancies, chimeric antigen receptor (CAR) T cells have emerged as a focus of modern immunotherapies across multiple medical fields. CAR T-cells are genetically engineered T lymphocytes that express chimeric antigen receptors (CARs) on their surface. Those receptors recognize antigens expressed by autoimmune or cancerous cells. Upon antigen recognition, lymphocytes release cytotoxic molecules such as granzyme, perforin, and cytokines, leading to apoptosis and lysis of targeted cells [1,2].

CARs consist of four main domains: an extracellular antigen-biding domain, a hinge or spacer region, a transmembrane domain, and one or more intracellular signaling domains [3]. The antigen-binding domain, which consists of a single-chain variable fragment (scFv), is the part of CARs responsible for recognizing targeted cells. Typically, CARs target extracellular autoimmune or tumor-associated antigens via scFvs, resulting in major histocompatibility complex (MHC)-independent T-cell activation [3,4]. The most important characteristic of the antigen-binding domain, determining CARs’ function, is its affinity. It must be high enough to provide antigen recognition, induce CAR signaling, and activate T cells, but at the same time not overly high so as to cause CAR T-cell exhaustion or toxicity [5,6]. The hinge or spacer region connects the scFv to the transmembrane domain. Its function is to provide flexibility to overcome steric hindrances, as well as sustain an appropriate length, allowing the antigen-binding domain to access the target epitope [7,8]. The length of the spacer domain also influences the immunological synapse formation by ensuring the appropriate intercellular distance [9]. The main function of the transmembrane domain is to anchor CAR to the T-cell membrane. In addition, these domains can influence the expression level of CARs or the transmission of ligand recognition signals to the intracellular cytoplasmic domain [10,11]. The intracellular signaling domain is a key component of CAR T-cells, crucial for their activation, persistence, and clinical effectiveness [11].

### 1.2. Five Generations of CAR T-Cells

**Figure 1:**
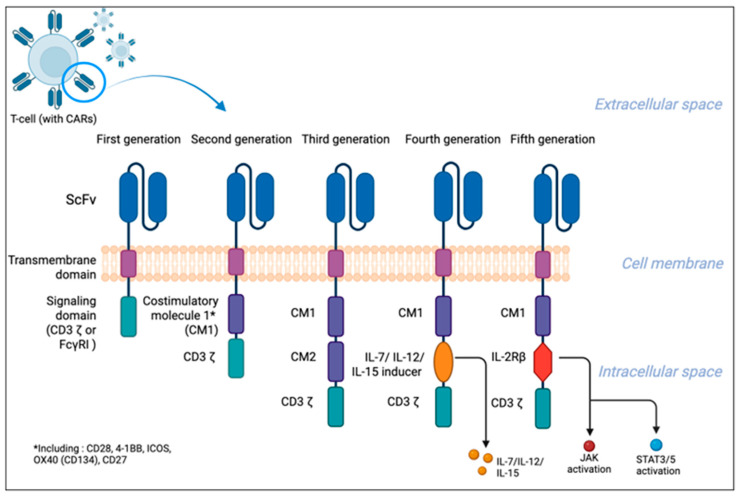
Evolution of five generations of chimeric antigen receptors (CARs). First-generation CARs contain a CD3ζ signaling domain; second- and third-generation CARs include one or two costimulatory molecules (CMs), e.g., CD28 and 4-1BB. Fourth-generation CAR T cells (TRUCKs) secrete cytokines (e.g., IL-7, IL-12, IL-15), while fifth-generation CAR T cells incorporate cytokine receptor signaling domains (e.g., IL-2Rβ) activating JAK/STAT pathways. Abbreviations: CARs—chimeric antigen receptors; CD—cluster of differentiation; CM—costimulatory molecule; ICOS— Inducible T-cell COStimulator; IL—interleukin; JAK—Janus kinase; FcγRI—Fc gamma receptor I; ScFv—single-chain variable fragment; STAT—signal transducer and activator of transcription; 4-1BB (CD137) — cluster of differentiation 137.

Based on the number and molecular structure of the intracellular domain, CAR T-cells can be divided into five generations [12,13]. Figure 1 illustrates the classification into five main generations.

First-generation CAR T-cells consist of an scFv, a hinge region, a transmembrane domain, and an intracellular signaling domain such as CD3-ζ or FcγRI, which acts as the main transducer of T-cell receptor (TCR) signaling and induces signaling cascades [14]. However, modest CD3 ζ-mediated activation led to insufficient therapeutic efficacy due to poor cytokine production and limited proliferation, which significantly altered the clinical outcomes [15,16]. To overcome these challenges, second-generation CAR T-cells were enriched with costimulatory domains such as CD28, 4-1BB (CD137), ICOS, OX40 (CD134), CD27, and others, which significantly improved T-cell proliferation, cytokine production, and persistence [17,18]. To further enhance T-cell function, third-generation CAR Ts combine multiple costimulatory domains (e.g., CD28 and 4-1BB) to enhance efficacy, and preclinical studies support their improved performance with a similar safety profile compared to earlier designs. A clinical trial of third-generation CAR T-cells targeting CD19 demonstrated greater expansion and persistence than second-generation CARs, although some data suggest that multiple costimulatory signals may promote lymphocyte exhaustion [19,20]. Fourth-generation CAR T-cells, also known as T cells Redirected for Universal Cytokine-mediated Killing (TRUCKs) or armored CAR T-cells, are an evolution of the second-generation design, enriched with mechanisms for precisely controlling the immune response. A key innovation is the ability to induce cytokine production (including IL-7, IL-12, and IL-15) directly within the tumor microenvironment, often via a module activated by the nuclear factor of activated T cells (NFAT) [21,22]. This process not only increases the cytotoxicity of the lymphocytes themselves but also recruits other immune cells, as well as promotes T lymphocyte infiltration within tissues, which is particularly important in the treatment of solid tumors [23,24]. To ensure safety, these systems are equipped with adjustable suicide genes that allow lymphocytes to be turned off in the event of severe complications [10]. The fifth generation of CAR T-cells are second-generation CARs containing an additionally shortened cytokine receptor domain (e.g., IL-2Rβ) fused to the biding site for the transcription factor STAT3. After antigen recognition, the JAK-STAT pathway is activated, in addition to the CD3ζ and costimulatory domains. This design more closely mimics natural T-cell activation by providing all three signals required for robust immune responses, leading to enhanced proliferation, persistence, and anti-tumor activity [25,26].

### 1.3. CAR T-Cell Production Process

**Figure 2:**
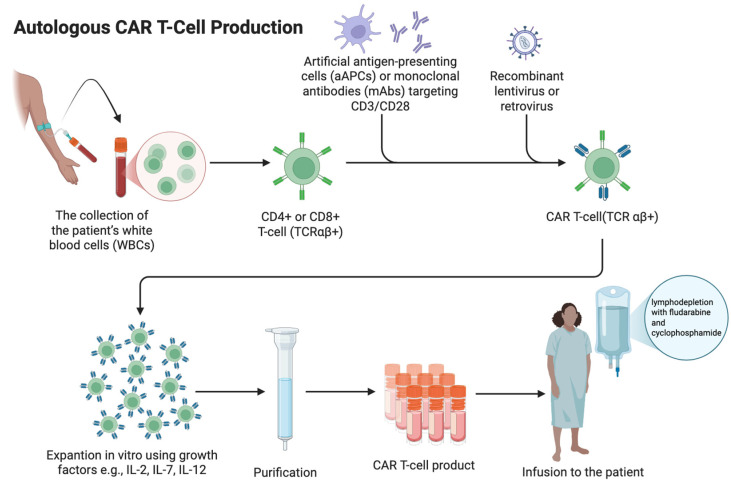
The process of autologous CAR T-cell production begins with collection of the patient’s white blood cells (WBCs). The isolated T cells are then activated in vitro and genetically modified using viral vectors to introduce the gene encoding the chimeric antigen receptor (CAR). The engineered CAR T-cells are subsequently expanded in vitro using cytokines and then cryopreserved. Before infusion, patients typically undergo lymphodepleting chemotherapy with fludarabine and cyclophosphamide, after which the CAR T-cells are administered and expand in vivo to exert their therapeutic effect. Abbreviations: CAR T-cell—chimeric antigen receptor T cell; CD—cluster of differentiation; IL—interleukin; TCRαβ+ — T-cell receptor alpha-beta positive; WBCs—white blood cells.

CAR T-cell manufacturing is a complex, multistep process (Figure 2).

CAR T-cell production can be either autologous or allogeneic. Allogeneic CAR T-cells are generated from healthy donor T cells that are genetically edited ex vivo using zinc finger nucleases (ZFNs) or transcription activator-like effector nucleases (TALENs) to disrupt TCR expression [27,28]. Alternatively, CRISPR/Cas9 enables CAR T-cell generation by inducing a targeted DNA double-strand break in T cells, allowing gene knockout (e.g., TCR) or precise insertion of the CAR transgene at defined genomic loci via DNA repair pathways [29]. Then, T cells are transduced with a viral vector (commonly lentiviral or retroviral) carrying the CAR construct during ex vivo culture. The cells are then expanded under controlled conditions, undergo quality testing, and are cryopreserved as an off-the-shelf product. In clinical practice, autologous CAR T-cell therapy remains predominant due to the absence of graft-versus-host disease (GVHD) risk, reduced risk of immune rejection, and the established manufacturing workflow [30]. The process of generating autologous CAR T-cells begins with the collection of the patient’s white blood cells (WBCs), after discontinuing immunosuppressive treatment at least three weeks prior to cell collection (except a low-dose prednisone) [31]. The collected T cells are then activated by artificial antigen-presenting cells (aAPCs) or monoclonal antibodies (mAbs) targeting CD3/CD28 [32]. T cells used in this process can be either CD4+ or CD8+, but CD8+ are more favorably used than autoreactive CD4+ cells despite the higher risk of CAR T-cell exhaustion [33]. The activated cells are then incubated with genetically modified viral vectors (lentiviral or retroviral) that contain the CAR gene. The vector delivers RNA encoding the CAR gene to the T cells. Then, the viral RNA undergoes reverse transcription into DNA, integrating it into the T-cell genome [34,35]. This allows T cells to express CARs on their surface. Genetically modified CAR T-cells are expanded in vitro using growth factors such as IL-2, IL-12, IL-7, IL-15, and IL-21 [34,36]. The final product is then cryopreserved before being administered to the patient [33]. In most cases, patients undergo lymphodepletion with fludarabine and cyclophosphamide prior to receiving the final product. This is the most common regimen used in clinical trials, but there is no specific standardization of this process. After administration of the CAR T product, these cells proliferate in the patient’s body, multiplying their quantity many times over and can persist for years, providing long-term remission [35].

### 1.4. Potential Applications of CAR T-Cell Therapy for the Treatment of Skin and Connective Tissue Disorders

In 2017, axicabtagene ciloleucel (axi-cel), a CAR T-cell therapy targeting the CD19 antigen on B cells, became the first product approved by the Food and Drug Administration (FDA) for the treatment of adult patients with relapsed or refractory (r/r) large B-cell lymphoma [37]. Since then, interest in CAR T-cell therapy has expanded into other fields of medicine, including dermatology and rheumatology. Current immunotherapies used to treat conditions such as systemic lupus erythematosus (SLE), systemic sclerosis (SSc), melanoma, and cutaneous T-cell lymphomas have limited efficacy and lead to disease progression, especially in severe systemic forms. Therefore, there has been a growing need for the development of novel therapies that more effectively target the underlying cause of the disease [38,39,40,41,42]. In recent years, CAR T-cell therapies targeting the CD19 antigen on B lymphocytes have been studied in the treatment of SLE and SSc. Additionally, oncological diseases including melanoma and primary cutaneous lymphomas are being studied with CAR T-cell therapies targeting specific antigens, e.g., GD2, cMET, and CD20 in melanoma and CCR4, TAG-72, and CD30 in the treatment of cutaneous T-cell lymphoma (CTCL) [43,44,45,46,47,48,49,50,51,52,53,54,55,56,57,58,59,60,61].

## 2. Methods

A literature review was conducted using electronic databases such as PubMed, Scopus, and Web of Science. Articles published within the last 5 years were searched using combinations of the following keywords: “CAR T-cell therapy,” “chimeric antigen receptor T cells,” “systemic lupus erythematosus,” “systemic sclerosis,” “melanoma,” “cutaneous lymphoma,” “Sjögren’s syndrome,” “pemphigus,” and “autoimmune diseases”. Articles not available in English or not directly related to the topic were excluded. The main objective of this paper is to identify diseases that may benefit from this treatment option and to assess its feasibility, efficacy, and safety based on evidence from recent case reports, case series, clinical trials, and preclinical studies.

Due to the narrative nature of this review, no formal systematic selection process or quality assessment was performed. The aim was to provide a comprehensive and up-to-date overview of emerging therapeutic applications rather than a quantitative synthesis of data.

## 3. CD19-Directed CAR T-Cell Therapy in Autoimmune Disorders

### 3.1. Systemic Lupus Erythematosus (SLE)

#### 3.1.1. Immunological Basis of Systemic Lupus Erythematosus (SLE)

SLE is a chronic autoimmune disease with heterogeneous clinical presentations affecting multiple organ systems [62,63]. Manifestations range from mild symptoms such as arthritis, fatigue, pleuritis, lymphadenopathy, and cutaneous lesions to severe, life-threatening complications including lupus nephritis, central nervous system (CNS) involvement, and systemic vasculitis. Severe disease occurs in a subset of patients and is associated with a poorer prognosis and increased mortality [63,64].

At the molecular level, the immunopathogenesis of SLE centers on a breakdown of immune tolerance with the generation of pathogenic autoantibodies targeting nuclear antigens. Central mechanisms include defective clearance of apoptotic cells and excessive neutrophil extracellular trap (NET) formation, leading to persistent antigenic stimulation and chronic inflammation. Dysregulation of innate and adaptive immunity is driven in part by sustained overproduction of type I interferons (IFN-α, IFN-β), which amplify autoreactive processes and contribute to an imbalance between T helper 17 (Th17) cells and regulatory T (Treg) cells. This imbalance supports pro-inflammatory responses and loss of self-tolerance, promoting autoantibody production by autoreactive B cells and immune complex-mediated tissue injury [65,66,67,68]. B cells are central to SLE pathogenesis not only through autoantibody production, but also via antigen presentation and cytokine secretion. Normally, autoreactive B cells are eliminated during central tolerance in the bone marrow and peripheral tolerance in secondary lymphoid organs. A breach of these checkpoints results in the survival and expansion of autoreactive clones. Phenotypic markers such as CD19, CD20, CD21, CD24, CD27, IgM, and IgD characterize B-cell differentiation stages, with CD19 present on nearly all B-cell stages and CD20 absent on pro-B cells and plasma cells—an important distinction for targeted therapies [69,70,71,72,73,74].

The treatment of SLE aims to control disease activity, prevent organ damage, and improve long-term outcomes and quality of life. Therapy is individualized according to disease severity based on organ involvement and includes immunosuppressive and biologic agents [75,76]. Hydroxychloroquine inhibits endosomal TLR7/9 signaling and reduces type I interferon production, remaining the cornerstone therapy with required ophthalmologic monitoring [77]. Glucocorticoids are widely used for rapid disease control during flares but are tapered to limit cumulative toxicity [78,79,80]. Immunosuppressants (methotrexate, azathioprine, mycophenolate mofetil, cyclophosphamide) inhibit lymphocyte proliferation. NSAIDs and low-dose aspirin provide symptomatic relief and thrombosis prevention [75,81,82]. Biologic therapies target key immune pathways. Belimumab, a monoclonal antibody against BLyS/BAFF, reduces B-cell survival and disease activity [83]. Rituximab, an anti-CD20 monoclonal antibody, depletes mature B cells but spares plasma cells, limiting efficacy [73,83,84,85]. Newer anti-CD20 agents, such as ocrelizumab and obinutuzumab, show mixed results [86,87,88]. Anifrolumab blocks IFNAR1, reducing type I interferon signaling, while voclosporin inhibits calcineurin and T-cell activation; however, remission remains uncommon [75,76]. Adjunctive therapies such as intravenous immunoglobulin and plasmapheresis may be considered in severe cases, though long-term benefit is limited [75,76].

Given the central role of B cells and the limitations of biological agents, CAR T-cell therapy has emerged as a promising approach for refractory SLE. CAR T-cells express a synthetic receptor targeting CD19, leading to selective B-cell lysis [2,23,24,25,26]. In SLE, CD19-directed CAR T-cells can deplete autoreactive B cells, including those resistant to anti-CD20 therapy, potentially enabling a deeper reset of immune system and sustained remission [43,72,73,89].

#### 3.1.2. Case Reports and Case Series

The early case reports and pilot studies Mougiakakos et al. (2021), Zhang et al. (2021), and Mackensen et al. (2022) demonstrated feasibility and clinical responses in refractory SLE, while studies published since 2023 confirmed efficacy and safety in larger, diverse cohorts [90,91,92].

In a case series by Jinhui Shu, Wei Xie et al., CD19-directed CAR T therapy (relma-cel) was evaluated in eight female patients aged 18–70 with active SLE following lymphodepletion with fludarabine and cyclophosphamide. Patients received a single infusion across four dose levels. Treatment led to marked clinical improvement, with substantial reductions in the Safety of Estrogens in Lupus Erythematosus National Assessment—Systemic Lupus Erythematosus Disease Activity Index (SELENA-SLEDAI) score, decreasing from 11.75 at baseline to 1.63 at 6 months. Six patients achieved a SELENA-SLEDAI score ≤ 4 within 3 months, including three complete clinical remissions, one as early as 1 month post-infusion. All patients met Systemic Lupus Erythematosus Responder Index (SRI) response criteria, although one experienced serological relapse at month three. Adverse events occurred in all patients but were predominantly grade 1–2, most commonly cytopenia (100%), cytokine release syndrome (CRS) (88%), and hypogammaglobulinemia (88%). No immune effector cell-associated neurotoxicity syndrome (ICANS) or significant organ toxicity was observed [49].

Pediatric application of anti-CD19 CAR T in SLE was reported by Krickau et al. in a 15-year-old girl with refractory class IV lupus nephritis requiring dialysis. Following lymphodepletion and CAR T-cell infusion, the patient showed improved renal function and was able to discontinue dialysis within 3 weeks. This was accompanied by rapid B-cell aplasia, normalization of complement levels, and anti-dsDNA seroconversion within 6 weeks. Over the 6-month follow-up period, proteinuria decreased to 3400 mg/kg/day.

Additionally, in a complex clinical case reported by Friedberg et al., anti-CD19 CAR T-cell therapy initially administered for refractory diffuse large B-cell lymphoma in a patient with coexisting SLE and antiphospholipid syndrome resulted in sustained oncologic remission and a complete immunological response. The treatment led to rapid and durable disappearance of all antiphospholipid antibodies, including lupus anticoagulant, anticardiolipin, and anti-β2 glycoprotein I antibodies, accompanied by normalization of ANA titers. Serological remission persisted throughout one year of follow-up, with no recurrence of thrombotic events or SLE activity [48].

#### 3.1.3. Cohort Studies

In a compassionate-use cohort by Taubmann et al. involving seven patients with severe refractory SLE and active lupus nephritis, a single infusion of autologous CD19 CAR T-cells after lymphodepletion resulted in a sustained immunological response. Complete peripheral B-cell depletion, normalization of complement levels, disappearance of anti-dsDNA antibodies, and achievement of Definition of Remission in SLE (DORIS) remission as well as low disease activity was achieved by all patients within a median follow-up period of 13 months. No ICANS was reported and CRS if observed was mild (grade I) [44].

A multicenter cohort by Müller et al. expanded the scope to 15 patients with severe autoimmune diseases including SLE, demonstrating drug-free remission in all SLE patients and proteinuria resolution after 3 months, normalization of disease activity scores, reconstitution of predominantly naïve B cells with complement level normalization and anti-dsDNA seroconversion. The therapy was well tolerated, with predominantly grade 1 CRS, hypogammaglobulinemia, and one case of pneumonia requiring hospitalization [43].

#### 3.1.4. B-Cell Maturation Antigen (BCMA) and CD19 CAR T-Cell Therapy

Emerging dual-target constructs combining B-cell maturation antigen (BCMA) and CD19 have demonstrated improved ability to eliminate long-lived plasma cells responsible for persistent autoantibody production.

In a phase I study conducted by Wang et al., bi-specific BCMA-CD19 CAR T-cells led to a substantial reduction in autoantibody levels, eradication of plasma cells, and sustained symptom- and medication-free remission (MFR) over a 46-month follow-up period in most treated patients with SLE and lupus nephritis [47].

Furthermore, a phase I study by Huang et al. evaluated sequential infusion of CD19 and BCMA CAR T-cells in 12 patients with refractory SLE. The Systemic Lupus Erythematosus Disease Activity Index 2000 (SLEDAI-2K) score decreased in all patients, from 18.3 to 1.5. All patients were able to discontinue SLE-related medications, including glucocorticosteroids. Low-level proteinuria persisted in some patients, possibly due to previous accumulated glomerular filtration impairment. The median follow-up was 118.5 days, and to date no SLE flares have occurred. Grade 1 CRS was the most common adverse event, and no cases of ICANS were reported. Recovery of B cells typically occurred by ~3 months post-infusion, often with a naïve phenotype suggestive of immune “reset,” while clinical benefit persisted [45].

Table 1 provides an overview of the key findings from the clinical cases and trials of CAR T-cell therapy in SLE.

### 3.2. Systemic Sclerosis (SSc)

#### 3.2.1. Immunological Basis of Systemic Sclerosis (SSc)

Systemic sclerosis (SSc) is a rare rheumatic disorder characterized by immune dysregulation, leading to widespread vascular damage and progressive fibrosis. It primarily affects the skin and connective tissue, but often involves multiple organs and systems, including the lungs, kidneys, heart, gastrointestinal tract, and musculoskeletal system. SSc is marked by the highest mortality rate among all rheumatic diseases [93,94].

Pathogenesis involves both innate and adaptive immune systems. Early disease events include microvasculopathy and immune activation, with T cells contributing to fibrosis through IL-4 and IL-13 production, endothelial cytotoxicity, and B-cell stimulation. B cells further drive disease via autoantibody production, secretion of pro-inflammatory cytokines such as IL-6, and antibody-dependent cytotoxicity, while regulatory B-cell function is impaired [95,96,97]. The presence of disease-specific autoantibodies, including anti-topoisomerase I (Scl-70), anticentromere (ACA), and anti-RNA polymerase III (anti-RNAPIII), reflects strong adaptive immune involvement [98]. Innate immunity also plays a key role, particularly plasmacytoid dendritic cells (pDCs), which are activated by CXCL4 and promote type I interferon (IFN-α) production, thereby linking innate and adaptive immune responses and sustaining disease progression [99]. Together, these mechanisms contribute to progressive fibrosis and vascular damage, which are hallmarks of SSc.

Elevated B-cell-stimulating factors and evidence of improved fibrosis following B-cell depletion in experimental models further support a central role for B cells in disease pathogenesis [100,101]. SSc shows dysregulated B-cell signaling with increased CD19 and reduced CD22 expression, driven by elevated B-cell activation factor (BAFF) levels that correlate with disease severity [97,102]. Based on these findings, CD20-targeted therapies such as rituximab have been evaluated, but they provide limited disease control in many patients [103]. In severe, refractory cases, autologous hematopoietic stem cell transplantation (auto-HSCT) may be considered, although its use is restricted by significant treatment-related mortality [104].

#### 3.2.2. Case Reports and Case Series

Case reports of CAR T-cells targeting CD19^+^ B cells suggest that this therapy can induce a deeper and better tolerated reset of the immune system in patients with SSc [43,50,51,52,53,54,55].

In a case series conducted by Pecher et al., five patients with severe life-threatening SSc received CD19-targeted CAR T-cell therapy. Four patients showed clinical improvement, including reduced skin involvement according to modified Rodnan skin score (mRSS), and fewer digital ulcers in one patient. All patients showed improvement in general physical condition along with a reduction in organ involvement, including enhanced lung function in 4/5 patients, reduced gastrointestinal involvement in one patient with severe weight loss, and stabilization of the cardiac disease in one patient, who experienced no new cardiac events. One patient with advanced disease and renal failure developed severe complications, including hemophagocytic lymphohistiocytosis (HLH) due to herpes simplex virus infection after an allergic reaction to acyclovir and massive CAR T expansion, ultimately resulting in death. Peripheral B cells were effectively depleted in all patients, with partial recovery in three cases after 3 months, and Scl70 autoantibodies temporarily declined. Mild CRS occurred in most patients and no neurotoxicity or major infectious complications were reported. The mean follow-up period was 250 days [50].

In another case series by Auth et al., six adult patients with severe diffuse SSc received CD19-targeted CAR T-cell therapy, resulting in significant clinical improvement and a good safety profile. Skin involvement improved in all patients (median 31% (≈8 points) mRSS reduction within 100 days), and three patients had a four-fold decrease in digital ulcers. Hand function and disability improved, with increased grip strength and a 36.6% faster task completion on the Moberg Picking-Up Test. Lung function remained stable for up to 600 days post-infusion, with a median 4% reduction in interstitial lung disease (ILD) extent on CT, mainly from decreased ground-glass opacities. Myocardial fibrosis remained stable, brain natriuretic peptide (pro-BNP) levels decreased in one patient, and renal function improved in one case, allowing reduced dialysis frequency (from 3×/week to 2×/week). Peripheral B cells were depleted within the first week and recovered in 2–6 months, with antinuclear antibody (ANA) titers declining 10-fold within 3 months. The therapy was well tolerated, with only mild to moderate CRS (grades 1–2), no neurotoxicity, and no need for additional immunosuppressive or antifibrotic therapy during the 487-day follow-up period [51].

Various case reports using CD19-targeted CAR T-cell therapy demonstrate clinical efficacy and a safety profile characterized by predominantly mild adverse events. Most patients experienced clinical improvement, regression of skin and visceral fibrosis, as well as seroconversion with disappearance of autoantibodies, without serious treatment-related complications; in most cases, only grade 1 cytokine release syndrome (CRS) was observed [53,54,55].

#### 3.2.3. Clinical Trials

Furthermore, in an early-phase clinical trial conducted by Wang et al., using TyU19, an allogeneic CD19 CAR T-cell therapy, two patients with refractory diffuse cutaneous systemic sclerosis (dcSSc) presented skin fibrosis improvement (mRSS scores dropped from 26 to 6 (S0102) and from 39 to 19 (S0103) by 6 months), improved lung and cardiac parameters (nearly complete high-resolution computed tomography (HRCT) lesion resolution with mild forced vital capacity (FVC) improvement, and cardiac magnetic resonance (CMR) showed reduction in edema/fibrosis), as well as marked reduction in anti-Scl-70 autoantibodies. No CRS or graft-versus-host disease (GVHD) was reported, and immunoglobulin levels were preserved. Patient condition remained stable during a 6-month follow-up period [52].

Additionally, in a clinical trial involving four patients diagnosed with SSc, CD19 CAR T therapy resulted in mRSS decreasing by a median of −9 points (IQR−10 to −7) in all patients within 6 months after administration. The level of disease-related autoantibodies decreased or disappeared, and there was a permanent loss of memory cells and pathogenic B cells [43].

In a controlled study of patients with systemic autoimmune diseases, including two patients with SSc, safety and CAR T/B-cell data were available for all eight patients, while clinical efficacy was assessable in five patients (follow-up ≥6 weeks). CAR T-cells expanded in all patients, leading to clinical response (including stable lung function in 1 patient with SSc), complete B-cell depletion within 10 days, and discontinuation of all immunosuppressive therapies. Treatment was well tolerated, with no grade 3–4 CRS, ICANS, or prolonged myelotoxicity. Adverse events were limited, including neutropenia, SLE exacerbation in one patient, and two cases of pneumonia (SARS-CoV-2 and CMV), which resolved with appropriate treatment. The follow-up period was 120 days [105].

We are currently awaiting official results from the Phase 1 Breakfree-1 study (NCT05869955), which enrolled 26 patients with SSc. Current information regarding the study results includes data from 19 SSc patients and shows improvement in lung function, interstitial lung disease (ILD) as well as clinically significant improvement in skin thickness. A median relative predicted forced vital capacity (pFVC) increase from baseline of 10% was seen at six months (*n* = 6) in subjects with ILD at baseline. Most of all, treatment-emergent adverse events (TEAEs), which occurred soon after infusion, were mild in intensity, and resolved rapidly with standard treatment. CRS in patients was mild and resolved within two days. Two patients experienced grade 3 ICANS events, which were transient and resolved completely within five days [106].

Table 2 represents the main results of the most important case reports, case series, and clinical trials regarding the efficacy and safety of CAR T therapy in the treatment of severe forms of SSc.

## 4. CAR T-Cell Therapy in Dermato-Oncology

### 4.1. Melanoma

#### 4.1.1. Therapeutic Targets of CAR T-Cell Therapy in Melanoma

Melanoma remains the deadliest form of skin cancer due to its high metastatic potential [107]. It originates from melanocytes located at the dermal–epidermal junction, which undergo uncontrolled proliferation [108]. Its incidence has increased in recent decades, and although melanoma accounts for only a small fraction of all skin cancers, it is responsible for over 80% of skin cancer-related deaths [109,110]. The risk group includes individuals with fair skin, a history of sunburn (especially during childhood), older adults, and those with a family history or prior history of skin cancer [111]. Standard therapies, such as chemotherapy, radiotherapy, and surgery, face limitations due to melanoma’s inherent resistance and tendency to recur [112]. This has prompted the search for new clinical and therapeutic approaches, with anti-PD-1 checkpoint blockade immunotherapy, and BRAF inhibitor-targeted therapy being the most advanced option [113,114].

Although CAR T therapy has shown significant efficacy in treating hematologic malignancies, the treatment of solid tumors presents greater challenges, such as limited tumor trafficking, limited infiltration, the presence of an immunosuppressive tumor microenvironment (TME), as well as adverse events associated with such therapies [114,115]. According to the 2009 American Joint Committee on Cancer (AJCC) staging system, stage IV, in which tumor cells metastasize to distant organs, is considered metastatic melanoma [116]. Melanoma cells metastasize primarily via the lymphatic route, but in some cases, the hematogenous route also appears to be involved [117].

Possible implications of CAR T-cell therapy include targeting antigens associated with cancer metastasis, such as angiogenic factors, extravasatory and intravascular factors (cadherins, integrins, immunoglobulin superfamily (IgSF), matrix metalloproteinases), factors responsible for leukocyte-tumor cell fusion and tumor cell embolism, cancer stem cells, and chemotactic molecules [40,118,119]. Current clinical trials of CAR T-cell therapy in melanoma target antigens such as GD2, CD20, and cMET [40,56,57,58]. GD2 is a disialoganglioside expressed in tumors of neuroectodermal origin and a tumor antigen of significant importance, whose increased expression has been reported in neuroblastoma, melanoma, retinoblastoma, gliomas, small-cell lung cancer, and sarcomas, including Ewing sarcoma, osteomas, and soft-tissue sarcomas [56]. CD20-positive cells have been reported within the tumor microenvironment of melanoma. Intralesional administration of the anti-CD20 monoclonal antibody rituximab has been associated with regression of metastatic melanoma in a single case report [57]. The mesenchymal–epithelial transition (MET) proto-oncogene encodes the hepatocyte growth factor receptor; a cell-surface protein tyrosine kinase found physiologically on epithelial cells of the liver, pancreas, prostate, kidney, muscle, and bone marrow. MET is also found in many solid tumors and is widely associated with cancer cell proliferation, invasion, and metastasis. High MET expression is associated with poor prognosis in breast cancer, and c-MET represents a potential therapeutic target in both breast cancer and metastatic melanoma [58].

#### 4.1.2. Results of Clinical Trials

Preclinical in vitro and in vivo murine studies demonstrated enhanced cytotoxicity, effective tumor cell eradication, tumor regression, and improved survival [40]. Building on these findings, Phase I clinical trials of CAR therapy in metastatic melanoma are currently ongoing, with results pending. In the phase 1 trial conducted by Garett et al., nine patients with GD2-positive metastatic melanoma, seven of which had the BRAF/MEKi mutation, received autologous GD2-specific CAR T-cell therapy. According to the results, 93% of CAR T products were successfully administered to the patients, and all patients showed some level of CAR T-cell expansion. Increased cytokine release (IL-6, IL-8, IFN-γ, TNF-α, GM-CSF) indicated CAR T-cell-induced immune activity. Moreover, no dose-limiting toxicities or severe adverse events (AEs) were noted, just as no neurotoxicity was observed. The main challenge was limited clinical efficiency—partial responses occurred only with concurrent BRAF/MEK inhibitors, and CAR T therapy alone showed minimal effect, requiring modifications before phase 2 trials. Even though CAR T-cells were detected in biopsies, tumor response was limited. Additionally, expansion of circulating myeloid-derived suppressor cells (MDSCs) post-treatment suggested a potential immune-suppressive response to CAR T therapy. The mean follow-up period was 42 days [56].

In another clinical trial, Aleksandrova et al. evaluated the feasibility and functionality of autologous anti-CD20 CAR T-cells in patients with stage III/IV melanoma. Patients were pre-treated with 60 mg/kg body weight cyclophosphamide (day −7 and day −6) and 25 mg/m^2^ body surface area fludarabine (day −5 to day −1) before intravenous infusion of MB-CART20.1 on day 0. Expansion of anti-CD20 CAR T-cells in patient blood, increased levels of INF-γ, as well as elimination of CD20+ tumor cells was noted during the study. However, differences in the levels of secreted cytokines and the degree of CAR T-cell amplification were observed. Notably, T-cell activation by CAR depended on the level of antigen expression. The more CD20 antigen there was on target cells, the greater the activation [57].

In a pilot phase I trail conducted by Shah et al., three patients with metastatic melanoma and metastatic triple-negative breast cancer (mTNBC) with at least 30% tumor expression of cMET received six infusions of autologous CAR T-cells without prior lymphodepleting chemotherapy. The cMET-directed CAR T-cell therapy was safe and feasible in patients with metastatic melanoma. Despite this, a clinical response to treatment was achieved in only four cases, which resulted in disease stabilization, and in five patients who underwent post-infusion biopsy, no CAR T-cell signal was detected in tumors. Eventually, all patients discontinued study participation due to disease progression and began alternate therapy or pursued hospice care. Most patients—6/7—experienced at least one adverse event that was related to study therapy. All AEs were grade 1 or 2 and medically manageable. One of the patients experienced CRS manifested by low-grade fever (maximum temperature 100.1 degrees Fahrenheit) and arthralgias which resolved within 24 h. Three clinically relevant adverse events occurred in more than one patient: anemia (*n* = 3), fatigue (*n* = 2), and malaise (*n* = 2). No serious adverse events or TLTs, as well as neurotoxicity, anaphylaxis, or allergic reactions, were observed [58].

#### 4.1.3. Preclinical Studies

In February 2024, a preclinical study evaluated CAR T-cells targeting tyrosinase-related protein 1 (TRP1) in rare, checkpoint inhibitor-resistant melanoma subtypes. Although TRP1 is mainly intracellular, a small fraction is expressed on the cell surface, with high levels in acral, mucosal (60%), and choroidal melanoma (90%). The therapy showed selective tumor targeting without harming normal TRP1-expressing cells. In vitro and in vivo murine studies demonstrated anti-tumor efficacy with no significant systemic toxicity or off-tumor effects [120].

Table 3 presents the most important results of studies on CAR T therapy in the treatment of melanoma.

### 4.2. Cutaneous T-Cell Lymphoma (CTCL)

#### 4.2.1. Potential Therapeutic Targets

Cutaneous T-cell lymphoma (CTCL) is a group of non-Hodgkin lymphomas derived from CD4-positive T cells, with mycosis fungoides (MF) and Sézary syndrome (SS) being the most common subtypes [121]. Due to the indolent nature of the early stages and lack of standardized treatment, CTCL has a poor prognosis [122]. Current therapeutic approaches include skin-directed therapies such as topical steroids, chlormethine gel, phototherapy or local radiotherapy, as well as systemic therapies including interferons, methotrexates, retinoids, total skin radiotherapy, chemotherapy, and allogeneic blood stem cell transplantation (allo-HCT). However, these treatments have limited efficacy in advanced disease stages [123,124]. Therefore, there is a need to develop new therapies that can effectively eliminate lymphoma cells.

CAR T-cell strategies in cutaneous T-cell lymphomas target several antigens. CD30, highly expressed in Hodgkin lymphoma and anaplastic large-cell lymphoma, is a validated therapeutic target, including brentuximab vedotin. CD70, a CD27 ligand involved in T-cell costimulation, is overexpressed in multiple hematologic malignancies and represents an emerging but less established target. One of the most studied CTCL-specific antigens to date is CCR4. This antigen is highly expressed in Sezary syndrome and mycosis fungoides, but it is not entirely specific for lymphoma cells. Its presence has also been demonstrated on Treg cells and some normal Th2 lymphocytes. It plays a crucial role in malignant lymphocyte chemotaxis. CCR4 is a clinically validated target of mogamulizumab. TAG-72 is mainly an epithelial tumor-associated glycoprotein, with limited hematologic expression, investigated primarily as an experimental or diagnostic target [59,60,61,125].

#### 4.2.2. Preclinical Studies

In a study by Evtimov et al., significantly higher levels of TAG-72 expression were found on the surface of circulating CD3+ and CD4+ T cells from CTCL patients compared to healthy donors. In vitro, CAR T-cells directed against TAG-72 effectively eliminated CD3+ TAG-72+ cells from the peripheral blood of CTCL patients, compared to control cells. Furthermore, the study demonstrated that CAR T-cells isolated from both CTCL patients and healthy donors demonstrated comparable functionality in in vitro assays, suggesting their therapeutic potential. In a mouse model transplanted with OVCAR-3 ovarian cells, CAR T-cells from CTCL patients eliminated tumor cells, achieving results comparable to CAR T-cells from healthy donors [59].

One of the latest preclinical studies conducted by To et al. tested the efficacy of CAR T-cell therapy directed against two different antigens. The authors developed and characterized three different CAR T-cell lines directed against TAG-72 and CD30 for the treatment of cutaneous T-cell lymphoma (CTCL). All three CAR T lines demonstrated high cytotoxic activity against CTCL tumor cells in vitro, reducing tumor mass and improving mouse survival. Additionally, the CAR T product demonstrated high specificity for cells expressing TAG-72 and CD30, minimizing damage to healthy cells. No serious side effects were observed in preclinical studies [60].

A study conducted by Watanabe et al. revealed promising results indicating that CCR4-CAR T-cells can provide a notable lytic activity against primary CTCL cells. Other key findings from their study included inhibition of helper and regulatory T-cell function, while sparing cytotoxic T lymphocytes and their anti-tumor activity, because the CAR T therapy specifically inhibits Th2, Th17 and Treg function, while saving CD8+ and Th1 T cells. Additionally, mogamulizumab-based CCR4-CAR T-cells induced superior anti-tumor efficacy and long-term remission in mice engrafted with human T-cell lymphoma cells [61].

#### 4.2.3. Clinical Trials

In a phase 1 study of six patients with CTCL, CCR4.CD30.CAR T therapy demonstrated a good safety profile but moderate clinical efficacy. The overall response rate (ORR) was 50% after 6 weeks of treatment, but no complete remissions (CR) were observed. A reduction in skin lesion severity was noted (median mSWAT reduction of 42.2%), but all patients required further treatment, and the median time to subsequent systemic therapy or death was relatively short (7.4 months). There were no treatment-related deaths, dose-limiting toxicities, or typical CAR T complications such as CRS or ICANS. The most common grade 3–4 adverse events were hematologic, primarily lymphopenia (in all patients), neutropenia, anemia, and thrombocytopenia, with relatively slow recovery. Two serious adverse events were also reported (including diverticulitis and severe neutropenia). The median follow-up was 26.0 months [126].

In a single-arm, open-label, phase 1 study by Iyer et al., 39 patients with relapsed or refractory peripheral T-cell lymphoma or cutaneous T-cell lymphoma received CTX130 therapy—CAR T-cells directed against CD70—after prior lymphodepletion with fludarabine 30 mg/m^2^ and cyclophosphamide 500 mg/m^2^ (intravenously daily for 3 days). CTX130 was administrated intravenously at dose levels ranging from 3 × 10^7^ CAR+ T cells (dose level 1) to 9 × 10^8^ CAR+ T cells (dose level 4). CRS was the most common adverse event, occurring in 26 (67%) of 39 patients (only one was a grade 4 dose-limiting toxicity at dose level 4). Grade 1–2 neurotoxic events were observed in 4 (10%) of 39 patients. The most common grade 3–4 adverse events were neutropenia (14 [36%]), anemia (11 [28%]), and thrombocytopenia (6 [15%]). Serious adverse events occurred in 25 (64%) patients, with CTX130-related serious adverse events in 14 (36%) patients, the most common related serious adverse event being cytokine release syndrome in 11 (28%) patients. Out of 39 patients, 18 had an objective response. The median patient follow-up was 7.4 months [127].

Table 4 summarizes the results of preclinical studies and clinical trials concerning CAR T therapy in the treatment of cutaneous T-cell lymphomas (CTCLs).

### 4.3. Cutaneous B-Cell Lymphoma (CBCL)

While CAR T therapy is being investigated in CTCL, data on its application in primary cutaneous B-cell lymphoma (PCBCL) remains limited. CAR T may be a promising therapeutic approach, but data specific to PCBCL is limited—most evidence comes from studies of systemic B-cell lymphomas and a few case reports with skin involvement [128,129,130]. This highlights a significant gap in knowledge and underscores the need for further preclinical and clinical studies to assess the safety and efficacy of CAR T therapy, especially for PCBCL. To date in B-cell non-Hodgkin lymphomas (NHLs), two CD19-specific CAR T-cells axicel and tisagenlecleucel are FDA-approved to treat relapsed and refractory DLBCL after at least two lines of systemic therapy [131,132]. One of the primary cutaneous B-cell lymphomas that could be cured with CAR T therapy is primary cutaneous diffuse large B-cell lymphoma, leg type (PCDLBCL, LT). Treatment of relapsed or refractory PCDLBCL, LT remains a challenge, and the National Comprehensive Cancer Network (NCCN) guidelines recommend second-line therapies typically used for systemic DLBCL [133]. Clinical trials such as ZUMA-7, TRANSFORM and BELINDA have confirmed the efficacy of CAR T-cell therapy as a second-line treatment for large B-cell lymphoma, with at least one case of PCDLBCL, LT reported in the ZUMA-7 study. The ZUMA7 and TRANSFORM studies showed benefits of axicel and lisocel respectively over standard of care, whereas BELINDA did not demonstrate superiority of tisagenlecleucel in the same setting [27,134,135]. These data suggest a possible evolution of the therapeutic approach in large B-cell lymphoma that may influence future treatment strategies for PCDLBCL, LT. Although the therapeutic rationale for using CAR T in PCBCL is strong, its clinical implementation remains theoretical at this stage and will require further validation.

## 5. Safety Concerns of CAR T-Cell Therapy

Despite its substantial therapeutic potential across both malignant and autoimmune diseases, CAR T-cell therapy is associated with a distinct and complex toxicity profile that may limit its broader clinical application. A comprehensive understanding of these adverse effects is essential to ensure treatment safety, optimize clinical outcomes, and guide appropriate patient selection. Importantly, most safety data derive from oncologic settings, whereas evidence in autoimmune diseases remains limited to small case series with a relatively short follow-up, necessitating cautious interpretation. Among the most frequently encountered and clinically significant complications is cytokine release syndrome, reflecting the potent immune activation intrinsic to CAR T-cell therapy.

### 5.1. Cytokine Release Syndrome (CRS)

CRS occurs in 42–93% of patients treated with CD19 CAR T therapies [136] and in 84–95% of those receiving BCMA CAR T products [137]. It arises from excessive cytokine and chemokine release, with IL-6 acting as a key mediator [138]. Although IL-6-blocking agents such as tocilizumab and siltuximab effectively alleviate CRS manifestations, they may be insufficient for preventing—and in some cases may even worsen—neurotoxicity (immune effector cell-associated neurotoxicity syndrome, ICANS) [137,139]. New approaches under investigation include preemptive use of IL-6–binding molecules (“IL-6 sponges”) and higher-frequency dosing of IL-1 inhibitors, such as anakinra, offering promising avenues for CRS mitigation. In addition, single-cell RNA sequencing (scRNA-seq) studies have revealed IFN-γ-driven inflammatory signatures and IL-1-associated resistance pathways, highlighting novel therapeutic targets. However, most of these strategies remain investigational, and their impact on CAR T-cell efficacy and persistence requires further validation [140,141].

### 5.2. Immune Effector Cell-Associated Neurotoxicity Syndrome (ICANS)

ICANS represents another major adverse effect, characterized by a wide range of neurological manifestations and typically appearing concurrently with, or shortly after, CRS [142]. Its underlying mechanisms are thought to involve disruption of the blood–brain barrier (BBB) due to endothelial injury and a systemic inflammatory environment, which together allow circulating cytokines and CAR T-cells to enter the central nervous system, ultimately leading to glial cell damage [143]. Mild ICANS is generally addressed with supportive measures and frequent neurological assessments. For moderate to severe cases, current ASCO and ASTCT recommendations designate corticosteroids—such as dexamethasone or methylprednisolone—as the primary treatment [144,145]. Tocilizumab, although beneficial for CRS, is not advised for isolated ICANS. High-throughput proteomic studies have highlighted IL-18 as a cytokine linked to ICANS onset, indicating that targeting the IL-18 pathway could potentially mitigate neurotoxicity [146]. Nevertheless, the efficacy of IL-18 blockade in ICANS prophylaxis or treatment has not yet been validated in preclinical or clinical trials. In parallel, next-generation CAR constructs are being engineered to reduce the risks of CRS and ICANS while enhancing tumor antigen specificity and T-cell activation.

### 5.3. Immune Effector Cell-Associated Hematotoxicity (ICAHT)

Growing clinical experience has increasingly emphasized cytopenias as a common and often subtle complication of CAR T therapy, now classified as immune effector cell-associated hematotoxicity (ICAHT) [147]. ICAHT is closely associated with both the depth and duration of neutropenia, with late ICAHT defined as neutropenia persisting beyond one month after infusion [148]. The CAR-HEMATOTOX model—incorporating measures of hematopoietic reserve such as baseline hemoglobin, platelet, and neutrophil counts, as well as ferritin and CRP levels—has proven effective in predicting delayed ICAHT and infection susceptibility [149]. As management strategies continue to advance, such predictive tools may support proactive approaches, including careful use of G-CSF and individualized anti-infective prophylaxis, enabling tailored interventions for early ICAHT [150]. In cases of extended cytopenia, previously collected autologous stem cells can be utilized, with successful augmentation reported following both CD19- and BCMA-directed CAR T therapy. For the minority of patients (<5%) with persistent, therapy-resistant late ICAHT, allogeneic hematopoietic stem cell transplantation (HSCT) remains the final therapeutic option [151]. Despite increasing recognition, standardized management strategies for ICAHT remain insufficiently defined.

### 5.4. On-Target Off-Tumor Toxicity (OTOT)

CAR T therapies directed against antigens that are also present on normal tissues can cause severe and sometimes fatal on-target, off-tumor toxicity (OTOT), a challenge most evident in the treatment of solid tumors. A notable example is the use of CD19 CAR T-cells, which effectively eliminate malignant B cells in ALL but also deplete normal B cells due to shared antigen expression [152]. Additionally, a subset of mural cells essential for maintaining BBB integrity express CD19 and may inadvertently be targeted, leading to BBB disruption and contributing to neurological toxicities [153]. The limited availability of truly tumor-specific cell-surface antigens—distinct neoantigens—complicates target selection. Most solid tumor targets are tumor-associated antigens (TAAs), such as EGFR, CAIX, and HER2, which are also present on healthy tissues [154]. Surface neoantigens are particularly uncommon in tumors with a low mutational burden [155]. Severe toxicities have been documented with CAR T therapies aimed at TAAs, including fatal lung injury with HER2-targeted CAR Ts, pulmonary toxicity with CEA-directed products, liver toxicity with CAIX-targeting CAR Ts, and skin toxicity with EGFR-directed therapies [156].

### 5.5. B-Cell Depletion

Recent evidence underscores that CD19 CAR T therapy not only eliminates malignant B cells but also induces profound depletion of normal B-cell populations across multiple tissue compartments. A landmark study by Tur et al. [73] demonstrated that CD19 CAR T-cells achieve deep, tissue-level B-cell clearance, with sequential ultrasound-guided lymph node biopsies from patients with autoimmune diseases revealing complete eradication of CD19+ and CD20+ B cells, disruption of follicular structures, and loss of follicular dendritic cells. Notably, whereas rituximab-treated individuals retained substantial lymph node B-cell reserves despite peripheral depletion, CAR T recipients exhibited uniform B-cell absence in both secondary lymphoid tissues and non-lymphoid organs—including the colon, kidney, and gallbladder—while plasma cells, T cells, and macrophages were preserved. This provides compelling in vivo evidence for a true immunologic “reset,” which may underpin the durable remissions observed after CD19 CAR T therapy. However, this same on-target mechanism contributes to B-cell aplasia, a clinically significant toxicity characterized by the loss of normal B cells and consequent hypogammaglobulinemia, often requiring immunoglobulin replacement. In addition, CAR T therapy is associated with bone marrow suppression leading to prolonged neutropenia, anemia, and thrombocytopenia. Together with CD4 lymphopenia and persistent cytopenias, these effects substantially heighten susceptibility to infections: bacterial infections are most common within the first month post-infusion, respiratory viral infections can occur for up to a year, and fungal infections develop in roughly 8% of patients [157].

### 5.6. Malignances Secondary to Therapy

The final, but most serious, complication of CAR T therapy is T-cell malignancies secondary to therapy. The role of CAR T-cells in this process is still being investigated, and 22 cases out of 27,000 treatments have been reported so far. This raise concerns that the genetic engineering process could cause malignant transformation of CAR T-cells, as viral insertion of the CAR gene, located near genetic regions that control T-cell growth, can cause malignancies through a process called insertional mutagenesis. However, thousands of patients have received CAR T-cell therapy for life-threatening hematologic malignancies, and these events are rare, and the benefits of CAR T-cell therapy outweigh the risks [158].

### 5.7. Fertility

Fertility considerations are particularly relevant in SLE, given that most patients are women of reproductive age. Conditioning regimens containing cyclophosphamide may contribute to cumulative gonadotoxicity, especially in patients with prior exposure. Future trials should systematically evaluate the impact of CAR T therapy and conditioning regimens on ovarian reserve and fertility, and alternative conditioning strategies (e.g., bendamustine-based regimens) may be explored to mitigate reproductive risk [159].

### 5.8. Overall Safety Considerations and Limitations

While CAR T-cell therapy represents a highly promising and potentially transformative approach, particularly in autoimmune diseases, current evidence remains limited in scale and duration. Most reported data derive from small, uncontrolled cohorts, and long-term safety—including risks of sustained cytopenias, infections, immune dysregulation, and secondary malignancies—remains incompletely characterized [157,158,160,161].

Importantly, the perception of a “favorable” safety profile in autoimmune indications should be interpreted with caution, as serious toxicities such as CRS, ICANS, and prolonged hematologic complications are well-established in oncologic settings and may similarly emerge with broader application. In addition, the extent to which immune reprogramming translates into durable, organ-level recovery varies across diseases and disease stages, particularly in the presence of irreversible tissue damage [46].

Importantly, emerging clinical observations suggest that despite profound immunologic effects, organ-specific responses may remain incomplete, particularly in advanced disease stages. For example, in a severe case of systemic lupus erythematosus, CAR T-cell therapy enabled dialysis independence and partial renal response; however, persistent proteinuria suggested irreversible glomerular damage, highlighting the limitations of immune reprogramming in established organ injury [46]. These findings underscore that while CAR T therapy may effectively control immune activity, it may not fully reverse structural damage.

Taken together, these considerations highlight that CAR T-cell therapy should currently be regarded as an investigational, high-risk but potentially high-reward strategy. Future studies with larger patient populations, longer follow-up, and systematic safety assessment are essential to define its true risk–benefit profile and optimal clinical positioning.

## 6. Future Perspectives

### 6.1. Future Perspectives in Autoimmune Diseases

Recent advances in CAR T technology and early clinical signals support a cautious but optimistic outlook for expanding CAR T approaches into dermatology. Novel delivery platforms—notably in vivo reprogramming using lipid-nanoparticle (LNP) RNA or cell-tropic viral vectors—promise to overcome the logistical bottlenecks of ex vivo manufacture and could make CAR T therapeutics more scalable and broadly accessible for autoimmune cutaneous diseases. Complementing delivery innovations are precision gene-editing strategies that enable more controllable, less genotoxic CAR designs (e.g., transient or regulatable CARs, safety switches, and “armored” constructs), which together reduce the barrier to testing CAR-based approaches outside oncology [162].

Emerging clinical and real-world observations indicate concrete dermatologic opportunities. Case reports and early autoimmune trials describe marked improvement or even remission of cutaneous autoimmune manifestations—for example, striking psoriasis resolution after CD19 CAR T administered for lymphoma and durable remissions in forms of cutaneous lupus in CD19-targeted programs—while phase-1 autoimmune programs (e.g., CD19 CAR T candidates) have reported encouraging safety and B-cell-depletion kinetics. These signals justify pilot studies in diseases with clear B-cell or autoantibody drivers (cutaneous lupus erythematosus, certain psoriasis endotypes, and antibody-associated dermatomyositis) and motivate exploratory work in Sjögren’s syndrome where glandular autoimmunity and B-cell pathology are prominent [163].

#### 6.1.1. Future Perspectives in Systemic Lupus Erythematosus (SLE) and Systemic Sclerosis (SSc)

Emerging clinical experience with CD19-directed CAR T-cell therapy in systemic autoimmune diseases highlights its potential to fundamentally redefine therapeutic paradigms, although disease-specific mechanisms and limitations remain distinct. In systemic lupus erythematosus (SLE), CAR T-cell therapy induces profound depletion of autoreactive B-cell compartments followed by reconstitution of a naïve, non-class-switched repertoire, consistent with a state of clonal remodeling and “immune reset” capable of attenuating interferon-driven immune activation [43,47,92]. In contrast, systemic sclerosis (SSc) represents a more complex therapeutic setting in which immune dysregulation is tightly linked to progressive fibrosis and structural tissue damage.

While early data in both diseases demonstrate rapid and sustained B-cell depletion with clinical improvement, the biological implications differ substantially. In SLE, clinical benefit appears to be closely associated with systemic immune reprogramming, raising the possibility of durable, treatment-free remission, particularly when intervention occurs early in the disease course [164,165]. In SSc, however, therapeutic efficacy may be constrained by the extent of established fibrosis, as CAR T-cell therapy primarily targets upstream immune drivers rather than irreversible tissue remodeling. Consequently, early intervention—prior to fixed fibrotic damage—may be critical to achieving meaningful clinical benefit [52].

Across both diseases, key unresolved challenges include the durability of response and the persistence of long-lived plasma cells and tissue-resident immune niches, which may enable relapse despite profound peripheral B-cell depletion [47,92]. These limitations provide a rationale for more refined targeting strategies, including dual CD19/BCMA constructs or approaches directed at autoreactive clones, as well as the need for biomarker-driven patient stratification. In SSc, this is particularly relevant for distinguishing immune-driven from fibrosis-dominant disease subsets.

Future research should therefore focus on defining the immunologic correlates of sustained remission, optimizing controlled immune reconstitution, and integrating molecular profiling with clinical outcomes. Ultimately, while CAR T-cell therapy holds promise as a mechanism-based intervention in both SLE and SSc, its long-term impact will depend on precise patient selection, early application, and the ability to align immune modulation with disease-specific pathophysiology.

#### 6.1.2. CAR T-Cell Therapy in Autoimmune Diseases: Emerging and Disease-Specific Implications

Beyond systemic autoimmune diseases, the application of CAR T-cell therapy in other immune-mediated conditions illustrates a spectrum of biological plausibility and therapeutic readiness.

In dermatomyositis, a disease characterized by autoantibody-defined heterogeneity and interferon-driven inflammation, CAR T-cell therapy may provide targeted modulation of pathogenic B-cell subsets. However, variability across molecular endotypes and the contribution of non-B-cell-mediated mechanisms suggest that therapeutic responses may be heterogeneous and require biomarker-guided stratification [166,167,168].

In Sjögren’s syndrome, the presence of ectopic lymphoid structures within exocrine glands introduces a tissue-centered dimension of disease pathogenesis. While CAR T-cell therapy may effectively deplete circulating B cells and modulate systemic immunity, its ability to eradicate tissue-resident B-cell populations and remodel glandular immune niches remains uncertain. This question is particularly relevant given the associated risk of B-cell lymphoma and the importance of preserving glandular function [169].

In contrast, psoriasis represents a predominantly T-cell-driven disease, and current evidence for CAR T-cell therapy is limited to isolated, hypothesis-generating observations [170,171,172]. The clinical relevance of B-cell depletion in this setting remains unclear, particularly in the context of highly effective targeted biologic therapies.

Pemphigus vulgaris (PV) is an autoimmune blistering disease caused by anti-Dsg1/Dsg3 antibodies, treated with steroids and immunosuppressants, characterized by significant morbidity and mortality due to treatment resistance and long-term complications [173,174]. It stands apart as a prototypical model for antigen-specific cellular immunotherapy. The development of desmoglein 3 (Dsg3)-targeting chimeric autoantibody receptor T (CAAR-T) cells enables precise elimination of autoreactive B-cell clones while sparing the broader immune repertoire [175,176,177,178]. This approach contrasts sharply with the broad immune reprogramming observed in CD19-directed therapies and highlights the potential of precision targeting in diseases with well-defined autoantigens. However, challenges such as epitope spreading, dual antigen involvement (Dsg1/Dsg3), and persistence of long-lived plasma cells remain to be addressed. Currently, as of December 2022, an open-label phase 1 study is undergoing to evaluate the safety and dosing of Dsg3 CAART in patients with pemphigus vulgaris with a predominance of anti-Dsg3 antibodies in the mucosa (mPV)(NCT04422912) [179].

Collectively, these observations suggest that the therapeutic role of CAR T-cell therapy across autoimmune diseases is highly context-dependent, ranging from mechanism-based systemic immune resetting to highly specific, antigen-directed interventions, with varying levels of clinical readiness.

#### 6.1.3. Long-Term Immunological Implications and Translational Challenges of CAR T-Cell Therapy

In addition to economic constraints, CAR T-cell therapy raises important long-term immunological concerns that remain insufficiently defined in autoimmune indications. Prolonged cytopenias, sustained B- or T-cell aplasia, and persistent immune modulation may impair host defense, vaccine responsiveness, and immune homeostasis, with a potential risk of secondary immune dysregulation [160,161]. While durable immune reprogramming underlies therapeutic efficacy, its long-term impact on immunocompetence requires careful evaluation.

These challenges highlight the need for more controllable and accessible therapeutic platforms. Advances in CAR design—including transient expression systems, inducible safety switches, and affinity tuning—aim to mitigate toxicity while preserving efficacy [180,181,182]. In parallel, emerging in vivo CAR T-cell engineering strategies, such as lipid nanoparticle-mediated mRNA delivery or viral vector-based approaches, may reduce reliance on complex ex vivo manufacturing and broaden clinical applicability [180,181,182]. Harmonized regulatory frameworks and structured long-term follow-up will be essential to define the safety, durability, and real-world effectiveness of CAR T-cell therapy in autoimmune and dermatologic diseases [160,161].

### 6.2. Future Perspectives in Oncology

#### 6.2.1. Melanoma

CAR T therapy in melanoma is constrained by the tumor microenvironment (TME), which limits infiltration, impairs effector function, and promotes T-cell exhaustion. Tumor heterogeneity and antigen loss facilitate immune escape, while suboptimal antigen selection increases off-tumor toxicity. Intrinsic resistance to apoptosis further compromises overall therapeutic efficacy.

##### The Tumor Microenvironment (TME)

The tumor microenvironment (TME) in melanoma is a complex three-dimensional network composed of tumor cells, cancer-associated fibroblasts (CAFs), tumor-associated macrophages (TAMs), blood vessels, the endothelium, the extracellular matrix (ECM), cytokines, and immune mediators. It creates major resistance to CAR T-cell therapy through three main mechanisms: immunosuppression (TGF-β, PD-L1, adenosine), physical exclusion (collagen, fibronectin, hyaluronan-rich ECM), and functional tumor adaptation (antigen loss such as Melan-A, VEGF/VEGFR2 signaling, and metabolic reprogramming via IDO) [183,184].

Immunosuppressive signaling pathways impair CAR T-cell activation and persistence. This phenomenon contributes to progressive T-cell exhaustion characterized by reduced effector function and diminished persistence within the tumor microenvironment. To overcome this, “armored CAR T-cells” have been engineered to secrete cytokines such as IL-12 and IL-15 or to neutralize suppressive factors like TGF-β, improving anti-tumor activity [185]. In addition, PD-1-modified Melan-A-targeted T cells and blockade of adenosine A2A signaling enhance CAR T-cell function by reversing checkpoint- and metabolically mediated inhibition [186,187,188]. VEGFR-2-targeted CAR T-cells expressing IL-12 further remodel the microenvironment by reducing suppressive myeloid cells [189]. IL13Rα2 has also emerged as a clinically investigated target linked to immunosuppressive signaling, although its role in melanoma remains under evaluation [190].

Inefficient tumor trafficking and infiltration are mainly driven by stromal architecture and dysregulated chemokine signaling. This leads to an immune-excluded tumor phenotype, where CAR T-cells remain confined to the tumor periphery without effective penetration into the tumor core. Chemokines such as CCL2–5 and CXCL9/10 correlate with T-cell infiltration, suggesting that modulation of chemokine–receptor axes may improve CAR T-cell homing [191,192]. Although temozolomide can increase CXCL9/10 expression, effective infiltration often requires additional disruption of the extracellular matrix. Combined enzymatic and pharmacological approaches, including collagenase-mediated stromal degradation, have been shown to enhance CAR T-cell penetration [193]. In addition, CAR T-cells engineered to express heparinase (HPSE) improve ECM degradation, resulting in increased intra-tumoral infiltration and anti-tumor activity [194].

Finally, melanoma cells undergo functional immune escape through antigen loss and pro-angiogenic signaling. Targeting pathways such as VEGFR-2 help reprogram the TME and improve immune activation. Overall, effective CAR T therapy in melanoma requires simultaneous targeting of immunosuppression, infiltration barriers, and tumor adaptation, often necessitating combination strategies for durable responses [189].

##### Antigen-Driven Limitations of CAR T-Cell Efficacy and Safety

One of the most important molecular factors influencing the effectiveness of CAR T therapy in the treatment of solid tumors is the selection of the specific antigen.

The same tumor may show different expressions of its antigens, which is often referred to as tumor heterogeneity. It enables selective survival and expansion of antigen-negative or antigen-low tumor subclones under CAR T-cell-mediated immune pressure, resulting in progressive immune escape and therapeutic failure [195,196]. Therefore, there is a risk that not all cancer cells express the target antigen. For this reason, various strategies to increase cancer detection by modifying CAR T-cells are being developed. One solution to this problem may be the engineering of CAR T-cells that simultaneously recognize two or more tumor antigens, which helps limit the risk of tumor cell escape through heterogeneity [197,198,199]. Another solution may be the strategy of pooled CAR Ts, which involves simultaneous or sequential administration of different CAR Ts directed against several antigens, which improves the range of tumor diagnosis and reduces the likelihood of the growth of antigen-negative clones. Moreover, the design of universal CAR T-cells, which separate the recognition domain from the signaling domain, allows for target switching and greater flexibility in recognizing multiple antigens without creating a new design for each target [199].

The loss of the targeted antigen by cancer cells is as big a challenge as the selection of the appropriate antigen. Immune editing induced by the therapy can lead to immune escape and tumor outgrowth. The previously mentioned multi-antigen CARs (dual CARs, or tandem CARs) increase the chances of recognizing cancer cells, even if some of them lose expression of specific antigens. However, beyond this, the use of “armored” CARs and stimulation of the immune response and epigenetic re-expression of antigens may also provide a proper solution. CAR T-cells designed to secrete cytokines or immunomodulatory factors—armored CARs—increase the activity of endogenous immune cells, which may counteract antigenic escape through the bystander killing effect. In turn, epigenetic modulators (e.g., DNA methylation inhibitors or HDACi) may restore the expression of antigens lost or silenced by cancer cells, potentially reducing the risk of antigen escape [199].

One of the major challenges with CAR T therapy is on-target/off-tumor toxicity. This occurs when CAR T-cells recognize and eliminate normal cells that also express the same antigen as the tumor, although to a lesser extent than cancerous cells. One solution to this problem is the design of “on-switch CARs,” which enables precise regulation of CAR T-cell activation. Their design requires the incorporation of an external activation molecule, which allows for control of the timing, location, and intensity of CAR T activity. This reduces adverse effects on healthy tissue. Additionally, by using various safety mechanisms, we can also minimize the toxicity of CAR T-cells. Suicide genes/safety switches (e.g., iCasp9) involve the introduction of genetic “death switches” that selectively disable CAR T-cells in the event of severe toxicity [199]. Affinity tuning involves modifying the affinity of the antigen-recognizing molecule so that CAR T-cells respond only to the high antigen expression typical of cancer cells. Low antigen expression in healthy tissues will not trigger CAR activation [156].

##### Resistance to CAR T-Cell-Induced Apoptosis in Melanoma

Melanoma resistance to CAR T-induced apoptosis is mainly driven by overexpression of antiapoptotic Bcl-2 family proteins and IAPs, which inhibit caspase activation and stabilize mitochondria [200,201]. Additional mechanisms include downregulation of death receptors (Fas, TRAIL-R) and reduced perforin/granzyme susceptibility [202]. This resistance can be mitigated by BH3 mimetics and IAP inhibitors, which sensitize cells to apoptosis [203]. Epigenetic agents (HDAC inhibitors, DNA demethylators) upregulate death receptors and proapoptotic genes [204]. CAR T engineering or combination with TRAIL agonists and NF-κB/ERK inhibitors further enhances tumor sensitivity and therapeutic efficacy [205].

##### Combination Strategies to Enhance CAR T-Cell Efficacy in Melanoma

Combination strategies are being explored to enhance CAR T-cell efficacy in melanoma. Oncolytic viruses, including Talimogene laherparepvec (T-VEC), can remodel the tumor microenvironment by inducing type I interferons, enhancing antigen release, reversing immunosuppression, and improving immune infiltration, although the timing is critical due to potential transient CAR T-cell inhibition [206,207,208,209]. Immune checkpoint blockade (CTLA-4 and PD-1 inhibitors) may restore CAR T-cell function by reversing exhaustion and inhibiting PD-1/PD-L1 signaling within the TME [210,211,212]. Additional approaches target immunosuppressive or pro-metastatic mediators such as MMPs or enhancing of anti-metastatic pathways. Overall, multimodal strategies are required to effectively reprogram the melanoma microenvironment [213,214,215].

#### 6.2.2. Cutaneous T-Cell Lymphoma (CTCL)

##### Antigen Heterogeneity in CTCL

In CTCL, malignant cells typically express CD3, CD4, and CD5, with frequent loss of CD7. Therapeutically relevant antigens include CD30, CCR4, CD25, and KIR3DL2. This contributes to high tumor heterogeneity and makes the selection of an appropriate therapeutic target challenging [216].

Due to tumor heterogeneity, targeting a single antigen may be insufficient to eliminate all lymphoma cells. Dual- or multi-specific CARs, which recognize two or more antigens simultaneously, can reduce tumor escape and improve coverage. To enhance both safety and efficacy, various logic gate systems have been developed. In the “HELP” logic gate system, the first antigen is expressed on both lymphoma and normal cells, while the second is tumor-specific. CAR T-cells are activated only after engagement of the second antigen, as the first CAR is truncated and lacks a costimulatory domain, preventing activation. In the “OR” logic gate system, CAR T-cells are activated upon recognition of either antigen, as each CAR contains a functional signaling domain. This increases the likelihood of tumor recognition and reduces antigen escape. For example, To et al. demonstrated that dual-targeting TAG-72 and CD30 CAR T-cells showed high specificity and minimized off-target toxicity. In the “NOT” logic gate system, CAR T-cells are inhibited when both antigens are engaged. Inhibitory CAR (iCAR) cells enable discrimination between tumor and healthy cells, improving safety [42,217].

##### Exhaustion of Normal T Lymphocytes in CTCL

Normal and malignant lymphocytes frequently share surface antigens such as CD3, CD4, CD5, CD7, CD30, and CCR4 [218], which creates a major limitation for CAR T therapy due to off-tumor toxicity. In CTCL, this is particularly critical, as targeting shared T-cell markers can lead to depletion of healthy T lymphocytes, resulting in immunosuppression, impaired immune surveillance, and increased susceptibility to severe infections [219]. Therefore, antigen selection remains a key determinant of both safety and efficacy in CAR T design. Several targets have been explored to improve specificity. CAR T-cells directed against TAG-72, CCR4, and CD70 have demonstrated selective cytotoxicity against lymphoma cells with relative sparing of normal T-cell populations, supporting their therapeutic potential [59,61,127]. However, limited antigen exclusivity remains a challenge. More promising targets include KIR3DL2, which is highly expressed in Sézary syndrome and mycosis fungoides but minimally present in healthy tissues, enabling improved tumor selectivity and reduced off-tumor toxicity [220,221]. An additional strategy exploits the clonal nature of CTCL through TRBC1 targeting. Since malignant T cells express either TRBC1 or TRBC2, while normal T cells express both, TRBC1-directed CAR T-cells can selectively eliminate the malignant clone while preserving a functional subset of normal T cells, thereby mitigating profound immunosuppression [222,223,224].

##### Fratricide of CAR T-Cells

Fratricide in CTCL refers to the self-elimination of CAR T-cells caused by the shared expression of target antigens on malignant and normal T lymphocytes (e.g., CD3, CD4, CD7, CD5). Because CTCL originates from T cells, CAR T constructs directed against T-cell markers can attack each other during manufacturing or after infusion. This reduces expansion, persistence, and overall therapeutic efficacy [225]. Strategies to overcome fratricide in CAR T therapy include selecting tumor-restricted antigens, genetically deleting the target antigen in CAR T-cells ex vivo, and using blocking antibodies during expansion to prevent scFv-mediated self-recognition. Antigen blockade can effectively reduce fratricide and enhance CAR T expansion; however, residual antibodies must be removed before infusion, as they may compete with CAR T-cells for tumor binding and reduce efficacy [226,227]. The extent of fratricide depends on the T-cell subset used for CAR T generation, therefore cells expressing the target antigen should be avoided. Alternatively, antigen expression can be silenced using genome-editing approaches such as CRISPR/Cas9, as demonstrated in CD7 CAR T systems, which prevents self-targeting while preserving cytotoxic function [228]. These engineering strategies collectively improve CAR T-cell viability, expansion, and therapeutic potential in T-cell malignancies.

##### Contamination of CAR T-Cell Product with Malignant T Lymphocytes

Contamination in CAR T manufacturing for CTCL occurs in the autologous setting when peripheral blood collected via leukapheresis contains a heterogeneous mixture of normal T lymphocytes and circulating malignant T cells. Because the starting material is patient-derived, complete separation of healthy and malignant T cells is not achieved. During ex vivo processing, all T cells are activated and transduced with the CAR construct. Consequently, lymphoma T cells may be inadvertently incorporated into the final product, resulting in contamination of the therapeutic CAR T population, with potential risks of reinfusion of malignant cells, enhanced tumor growth, and treatment failure [229]. Given that CTCL originates from the CD4+ T-cell subset, circulating tumor T cells can be collected during apheresis and transduced alongside healthy T cells during CAR production [230]. The higher the tumor burden in the blood, the greater the risk of CAR T product contamination, particularly in patients with advanced disease [42]. Malignant cells can then proliferate during ex vivo culture, potentially enhancing tumor growth and masking tumor antigens from the action of healthy CAR T-cells [158,231]. A possible solution to this problem is the use of allogeneic T lymphocytes to produce CAR T-cells. However, it should be remembered that a possible complication of this treatment is the occurrence of graft-versus-host disease (GVHD). Therefore, to minimize this risk, a donor with the closest human leukocyte antigen (HLA) match to the recipient should be selected. HLA matching, expansion time, and cell persistence are currently the focus of intensive research aimed at developing a truly universal therapy [42,232,233,234,235]. The second option is to generate CAR T-cells from a subset of disease-neutral T cells, i.e., purify and utilize the CD8+ population. To effectively implement this approach, a purification protocol is needed to prevent the incorporation of any malignant CD4+ T cells into the final therapeutic cell product [42,59]. Studies indicate that patients with mycosis fungoides tumors who have a higher number of infiltrating CD8+ lymphocytes have a better prognosis compared to patients whose lymphoma cells are predominantly CD4+ lymphocytes [236]. This suggests the potential efficacy of CD8+ CAR T-cell therapies. Both CD4+ and CD8+ CAR T-cells could eliminate tumor cells, but CD4+ cells also play a supportive role, increasing proliferation, survival, and a long-lasting CD8+ anti-tumor response through cytokine production and supporting the development of memory cells [237]. The highest efficacy is observed when using a combination of CD4+ and CD8+ CAR T-cells, particularly when the cells are derived from naive or central memory CD8+ populations, which are characterized by higher activity and longer survival [238]. However, a significant limitation remains the quality of autologous T lymphocytes collected from patients, which often demonstrate lower proliferation capacity than cells from healthy donors, indicating the need to optimize CAR T culture and production conditions to increase the efficacy of therapy in CTCL [239].

Table 5 summarizes the most important challenges of CAR T therapy and how to overcome them.

### 6.3. Production Costs, Availability, Regulatory Constraints of CAR T Therapy

CAR T-cell therapy is classified in the European Union as an advanced therapy medicinal product (ATMP). Autologous manufacturing is currently the standard clinical approach, which requires individualized leukapheresis, viral transduction, ex vivo expansion, and rigorous quality control, resulting in a vein-to-vein time of several weeks and treatment costs often exceeding €300,000 per patient. The above production requirements lead to a centralized production model that limits access exclusively to specialized, certified centers. Consequently, patients with advanced disease may need to travel, sometimes internationally, to receive treatment, further increasing costs of therapy and adding logistical as well as clinical burdens. Although the regulatory framework ensures safety and efficacy, it also extends implementation timelines due to stringent approval pathways and long-term pharmacovigilance requirements [243,244,245].

To address these challenges, several strategies are being developed, including decentralized or point-of-care manufacturing, automated closed-system production, and supply chain optimization to reduce production time. In addition, managed entry agreements, outcome-based reimbursement models, and national CAR T referral networks are increasingly implemented in Europe to improve affordability and equitable access while maintaining regulatory standards and clinical safety [246,247].

## 7. Conclusions

CAR T-cell therapy represents a breakthrough in modern immunotherapies. Its application may extend beyond hematological diseases to include severe, refractory autoimmune disorders such as systemic lupus erythematosus and systemic sclerosis, offering hope to patients unresponsive to standard immunosuppressive treatment options. Furthermore, CAR T therapy may be beneficial in advanced solid tumors such as melanoma, as well as cutaneous T-cell and B-cell lymphomas. Although CAR T therapy may represent the therapeutic future for the above-mentioned diseases, results from clinical trials, case series, and case reports on CAR T therapy are based on small and selected patient groups. Therefore, these data do not clearly determine the efficacy, long-term clinical benefits, or safety profile of the therapy, including severe neurological complications such as ICANS. Further multicenter clinical trials involving larger, more randomized patient groups and multi-year follow-up are necessary to determine the future of CAR T therapy beyond hematological diseases.

## Figures and Tables

**Table 1:** Clinical outcomes of CD19 CAR T-cell therapy in patients with SLE.

Author	Year	Number of Patients	Age (Years)	Sex	SLEDAI	Organ Involvements	Previous Treatment	CAR T-Cell Protocol	Response to CAR T-Cell Therapy	Complications
Case series and case reports										
Shu J., Xie W., et al. [49]	2025	8	18–70	8 F	NR	Kidney, blood	GCS, AZA MMF, MTX, CYC, CS, TAC, LEF	Flu 25 mg/m^2^ IV (days −5 to −3), Cy 1 g/m^2^ IV (day −3), CD19 CAR T-cells at four DLs: 25 × 10^6^ cells (*n* = 3), 50 × 10^6^ cells (*n* = 2), 75 × 10^6^ cells (*n* = 2), and 100 × 10^6^ cells (*n* = 1)	SELENA-SLEDAI ≤ 4 within 3 months in 6/8 cases, 4/8 met DORIS remission criteria, no symptoms with normalized complement and autoantibody levels at 12-month follow-up, 1/8 had serological relapse at 3 months	AEs mostly G1–2 (8/8); cytopenia (8/8), CRS (G1) (7/8), hypogammaglobulinemia (7/8); no ICANS or hepatic/renal toxicity; ICAHT ≤ G1 (7 pts)
Krickau et al. [46]	2024	1	15	F	23	Severe kidney disease requiring dialysis	HCQ, AZA, MMF, BEL	Flu 25 mg/m^2^ IV (days −5 to −3), Cy 1 g/m^2^ IV (day −3), CD19 CAR T ~1.1 × 10^6^/kg on day 0	Dialysis-free after 3 weeks, eGFR improved from 8 to 42 mL/min/1.73 m^2^, normalization of complement levels, anti-dsDNA seroconversion within 6 weeks, proteinuria decreased to 3400 mg/kg/day during 6-month follow-up	CRS G1—managed with tocilizumab, no ICANS
Fried berg et al. [48]	2024	1	65	F	NR	Blood, bone marrow	VKA, HCQ, GCS	Flu 25 mg/m^2^ IV (days −5 to −3), Cy 1 g/m^2^ IV (day −3), CD19 CAR T ~1.1 × 10^6^/kg on day 0	Complete B-cell aplasia, serological remission and no SLE activity for 12 months during follow-up	CRS G1, treated with tocilizumab and dexamethasone; ICANS G4 treated with methylprednisolone
Cohort studies										
Taubmann et al. [44]	2023	7	19–39	6 F 1M	NR	Kidney, heart, lungs, pleura, joints, skin, muscles and bone marrow	HCQ, AZA, MMF, BEL	Flu 25 mg/m^2^ IV (days −5 to −3), Cy 1 g/m^2^ IV (day −3), CD19 CAR T ~1.1 × 10^6^/kg on day 0	100% DORIS remission (median follow-up of 13 months), median B-cell aplasia 120 days	CRS (mostly G1)
Müller et al. [43]	2024	8	18–38	7 F 1M	Varied by disease	Skin, kidneys, lungs, heart, joints, bone marrow	GCS, HCQ, MMF, MTX, RTX, NIN, TOC, CYC	Flu 25 mg/m^2^ IV (days −5 to −3), Cy 1 g/m^2^ IV (day −3), CD19 CAR T ~1.1 × 10^6^/kg on day 0; patient 14: 50% dose	Complete B-cell aplasia in the mean follow-up of 15 months; durable remission, SLE patients achieved SLEDAI scores of 0	CRS (1/8 G2); patient 8 hospitalized for pneumonia (resolved with antibiotics), other infections mild (mostly URTIs); hypogammaglobulinemia, no ICANS
BCMA and CD19 CAR T-cell therapy—phase I clinical studies										
Wang et al. [47]	2024	12	NR	NR	NR	Kidney, joints, skin, heart, lungs	HCQ, GCS, CYC, MMF, TAC, RTX,	3 × 10^6^/kg cCAR T	Plasma cells eradicated <1 month, C3/C4 normalized ≤ 21 days, several patients with >1 year drug-free remission, renal improvement <6 months, patients achieved symptom-free remission and MFR with post-cCAR follow-up to 46 months	Mild CRS, normal immune recovery by ~150 days, no ICANS
Huang et al. [45]	2023	12	NR	NR	18.3	Kidney, lungs, joints, skin, bone marrow, muscles	GCS, MMF, MTX, HCQ, CYC, TCZ, RTX, NIN BEL, TET	3/12 received 1 × 10^6^/kg CD19 CAR T-cells and BCMA CAR T-cells; 9/12 received 2 × 10^6^/kg CD19 CAR T-cells and BCMA CAR T-cells 2 × 10^6^/kg	SLEDAI-2K score decreased in all patients, from a mean of 18.3 to 1.5 [median follow-up of 118.5 (45–524) days]	12/12 CRS G 1 (fever), no ICANS; hematologic toxicity in 12 patients, 4 infections (COVID-19 *n* = 2, GI *n* = 1, pulmonary *n* = 1) fully resolved within 6 months

Abbreviations: AEs—adverse events, AZA—azathioprine, BCMA—B-cell maturation antigen, BILAG—British Isles Lupus Assessment Group, BEL—belimumab, CAR T—chimeric antigen receptor T cell, CD19—cluster of differentiation 19, CRS—cytokine release syndrome, CS—ciclosporine, CYC/Cy—cyclophosphamide, DLs—dose levels, F—female, Flu—fludarabine, GCs—glucocorticoids (steroid medications used for immunosuppression and inflammation control), HCQ—hydroxychloroquine, ICAHT—immune effector cell-associated hematotoxicity, ICANS—immune effector cell-associated neurotoxicity syndrome (neurotoxicity complication that may occur after immune effector cell therapies), IV—intravenously, LD—lymphodepletion, LEF—leflunomide, LLDAS—Lupus Low Disease Activity State, M—male, MMF—mycophenolate mofetil, MFR—medication-free remission, mRSS—modified Rodnan skin score, MTX—methotrexate, NIN—nintedanib, NR—not reported, RTX—rituximab, SELENA-SLEDAI—Safety of Estrogens in Lupus Erythematosus National Assessment—Systemic Lupus Erythematosus Disease Activity Index, SLE—systemic lupus erythematosus, SLEDAI / SLEDAI-2K—Systemic Lupus Erythematosus Disease Activity Index, TAC—tacrolimus, TET—tetracycline, TCZ/TOC—tocilizumab, URTIs—upper respiratory tract infections, VKA—vitamin K antagonists.

**Table 2:** Clinical outcomes of CD19 CAR T-cell therapy in patients with SSc.

Author	Year	Number of Patients	Age (Years)	Sex	mRSS	Organ Involvements	Previous Treatment	CAR T-Cell Protocol	Response to CAR T-Cell Therapy	Complications
Case reports and case series										
Pecher et al. [50]	2025	5	42–68	M:1F:4	7–32	Skin, lungs, heart, gastrointestinal tract, kidneys	MTX, MMF, HCQ, CSA, CYC, RTX, NIN, TCZ, HSCT	Flu 25 mg/m^2^ IV (days −5 to −3), Cy 1 g/m^2^ IV (day −3), CD19 CAR T ~1.1 × 10^6^/kg on day 0	mRSS reduction in 5/5, ulcers reduced in 1/5, lung (4/5) and heart function improvement (1/5), weight gain (1/5), B-cell depletion by day +7, transient autoantibody decline, no disease flares during 250 days of follow-up	CRS G1 in 4/5 cases; HLH in 1/5 patients resulting in death
Auth et al. [51]	2024	6	36–53	M:4F:2	17–35	Skin, lungs, heart, kidneys	GCS, MMF, MTX, HCQ, CYC, TCZ, RTX, NIN	Flu 25 mg/m^2^ IV (days −5 to −3), Cy 1 g/m^2^ IV (day −3), MB-CART19 ~1.1 × 10^6^/kg on day 0	mRSS reduction in 31% (~8 points) by day 100, digital ulcers reduction ~4× in 50%; hand function and grip strength improvement, ILD modestly reduced, NT-proBNP decrease in 3/6, B-cell depletion, autoantibody decrease and disease stabilization during 487-day follow-up	CRS G1 in 3/6 and G2 in 2/6, hypogammaglobulinemia in 6/6, 4/6 patients required IVIG replacement therapy
Merkt et al. [53]	2025	1	38	F:1	22	Skin, lungs, heart	CYC, MMF, NIN	Flu 30 mg/m2 IV (days −4, to −2), Cy 500 mg/m2 IV (days −4 to −2), CD19. CAR T- cells 400 × 10^6^ (5 × 10^6^/kg of body weight) IV on day 0; NIN was continued throughout the 2-year observation period	Overall serological remission and major improvement of SSc-ILD and fibrosis during 2-year follow-up: mRSS reduction by 59%, dyspnea improved with FVC/DLCO increase, CT- reduction in ground-glass and fibrosis, cardiac/inflammatory markers normalized, CAR T-cells persisted >24 months, B cells and anti-Scl-70 remained absent	CRS G1
Claus et al. [55]	2024	1	NR	NR	NR	Lungs	RTX, TOC, NIN, MMF, GCS	NR	Lung function and ILD regression, mRSS was reduced by ~50%, autoimmune markers, including circulating immune complexes, normalized, clinical improvement persisted up to 11 months	None
Bergmann et al. [54]	2023	1	60	M:1	20	Skin, lungs, heart	MTX, MMF	Flu 12.5 mg/m^2^ (days −5 to −3), Cy 500 mg/m^2^ (on day −3), 1 × 10^6^ CAR T-cells/kg on day 0	Raynaud’s improved, skin fibrosis reduced, lung function preserved (DLCO +20.4%), cardiac function stable (EF) with improved PASP and RA area, ANA abolished, RP11 autoantibodies undetectable at 3–6 months, sustained clinical improvement during 6-mont follow-up	CRS G1
Clinical trials										
Müller et al. [43]	2024	4	36–60	M:3 F:1	18.8–30.8	Skin, kidneys, lungs, heart, joints	GCS, HCQ, MMF, MTX, RTX, NIN, TOC, CYC	Flu 25 mg/m^2^ IV (days −5 to −3), Cy 1 g/m^2^ IV (day −3), MB-CART19 ~1.1 × 10^6^/kg on day 0; patient 14 received 50% reduced dose	EUSTAR decreased by −4.2 and mRSS by −9 after ≥6 months, autoantibodies decreased or became undetectable, depletion of memory/pathogenic subsets of B cells, clinical and immunological improvement during 15-month follow-up	CRS G1 in 3/4; hypogammaglobulinemia (no information regarding IVIG replacement therapy)
Wang et al. [52]	2024	2	45- 56	M:2	26–39	Skin, lungs, heart, gastrointestinal tract	GCS, CYC, HCQ, MMF, TAC, TZC, BLM, RAPA, MSC	Flu 25 mg/m^2^ IV (days −5 to −3), Cy 300 mg/m2/day IV (days –5 and –4), CAR-positive TyU19 cells 1 × 10^6^/kg IV on day 0	ACR-CRISS ≥ 0.996 within 1–2 months and sustained to 6-month follow-up, mRSS reduction and improved skin elasticity, regression of lung and cardiac fibrosis, anti-Scl-70 levels significantly decreased, with near-complete elimination in one case	None

Abbreviations: ANAs—antinuclear antibodies, ACR-CRISS—American College of Rheumatology—Combined Response Index in Systemic Sclerosis, BLM—belimumab, CAR T—chimeric antigen receptor T cell, CD-19—cluster of differentiation, CSA—ciclosporin A, CT—computed tomography, CRS—cytokine release syndrome, CYC/Cy—cyclophosphamide, DLCO—diffusing capacity of the lungs for carbon monoxide, EF—ejection fraction, EUSTAR—European Scleroderma Trials and Research group, F—female, Flu—fludarabine, FVC—forced vital capacity, G—grade, GCS—glucocorticoids, GI—gastrointestinal, HCQ—hydroxychloroquine, HLH—hemophagocytic lymphohistiocytosis, HSCT—hematopoietic stem cell transplantation, ILD—interstitial lung diseases, IQR—interquartile range, IV—intravenously, IVIG—intravenous immunoglobulin, M—male, MB-CAR T—Mesothelin/B-cell Maturation Antigen Chimeric Antigen Receptor T cell, mRSS—modified Rodnan skin score, MMF—mycophenolate mofetil, MTX—methotrexate, MSC—mesenchymal stem cell transplantation, NIN—nintedanib, NR—not reported, NT-proBNP—N-terminal pro-B-type Natriuretic Peptide, NYHA—New York Heart Association, PASP—pulmonary artery systolic pressure, RA—right atrium, RAPA—rapamycin, RP11—RNA Polymerase III subunit 11, RTX—rituximab, Scl-70—topoisomerase I antibody, SSc—systemic sclerosis, TAC—tacrolimus, TCZ/TOC—tocilizumab.

**Table 3:** Results of clinical trials and preclinical studies using CAR T-cell therapy in metastatic melanoma.

Author	Year	Clinical trial ID	Number of Patients	Sex	Targeted Antigen	CAR T-cell Protocol	Response to CAR T-Cell Therapy	Complications
Clinical trials								
Gargett et al. [56]	2024	ACTRN12613000198729	9	NR	GD2	1 × 10^6^ GD2-CAR T-cells/kg on day 0; BRAF and MEK inhibition (dabrafenib and trametinib; started 7 days prior to infusion and continued for total of 28 days)	CAR T-cells were detected in tumor biopsies; however, tumor response was limited—most patients had disease progression or transient stabilization	Mild AEs including rash, fever, diarrhea, and anorexia; no neurotoxicity observed
Aleksandrova et al. [57]	2024	NCT03893019	3	F:2 M:1	CD20	Cy 60 mg/kg (day −7, −6) and 25 mg/m^2^ Flu (day −5 to −1); MB-CART20.1 on day 0 (the largest clinical dose 1 × 10^7^/kg)	All CAR T products induced T-cell activation, cytokine levels and CAR T expansion varied between patients, activation was dependent on target antigen expression level	NR
Shah et al. [58]	2023	NCT03060356	3	F:2 M:1	cMET	Six infusions (1 × 10^8^ T cells/dose) of CAR T-cells without lymphodepleting chemotherapy	Disease stability in 4/7, progression in 3/7, CAR T mRNA detected in peripheral blood of all patients, post-infusion biopsy showed no intra-tumoral CAR T signal in 5 cases (3 had paired tumor samples) IHC: ↑CD8, ↑CD3, ↓pS6, ↓Ki-67	G1 or 2 toxicities in 3/3 (anemia, fatigue, malaise), CRS G1 in 1/3
Preclinical studies								
Jilani et al. [120]	2024	NR	NR	NR	TYRP1	3.5 × 10^6^ CAR T-cells/kg or 7 × 10^6^ CAR T-cells/kg	Cytotoxicity against TYRP1-high melanoma cells, tumor regression and improved survival signals	No severe systemic toxicity

Abbreviations: AEs—adverse events, BRAF—B-Raf proto-oncogene, serine/threonine kinase, CD20—cluster of differentiation 20, cMET—cellular mesenchymal–epithelial transition factor, CRS—cytokine release syndrome, F—female, Cy—cyclophosphamide, Flu—fludarabine, G—grade, GD2—disialoganglioside GD2, GD2-CAR T—disialoganglioside GD2 Chimeric Antigen Receptor T cells, IHC—immunohistochemistry, M—male, MB-CAR T—Mesothelin/B-cell Maturation Antigen Chimeric Antigen Receptor T cells, MEK—Mitogen-Activated Protein Kinase Kinase, mRNA—messenger Ribonucleic Acid, NR—not reported, pS6—phosphorylated ribosomal protein S6, TYRP1—tyrosinase-related protein 1.

**Table 4:** Results of preclinical studies and clinical trials using different CAR T-cell therapies in CTCL.

Author	Year	Number of Patients	Sex	Targeted Antigen	CAR T-cell Protocol	Response to CAR T-Cell Therapy	Complications
Preclinical studies							
To et al. [60]	2025	NR	NR	TAG-72 and CD30	NR	CAR T lines showed potent, specific cytotoxicity against CTCL cells reduced tumor burden improved mouse survival	No serious side effects were noted
Evtimov et al. [59]	2024	NR	NR	TAG-72	CAR T group: 2 × 5 × 10^6^ cells IV (5-day interval); control: non-transduced T cells at matched dose and schedule	CTCL CD3+/CD4+ T cells exhibited elevated TAG-72 expression anti-TAG-72 CAR T cells selectively eradicated TAG-72+ cells in vitro	NR
Watanabe et al. [61]	2024	NR	NR	CCR4	0.5 × 10^6^ CCR4-CAR–positive T cells or untransduced T cells 7 days after tumor cell inoculation	Potent cytotoxicity selective CCR4+ cell depletion Th2/Th17/Treg suppression superior efficacy with durable remission in murine models	NR
Clinical trials							
Iyer et al. [127]	2024	39	F: 21 M:18	CD70 (CTX130)	Flu 30 mg/m^2^ + Cy 500 mg/m^2^ × 3 days; CTX130 infusion: 3 × 10^7^–9 × 10^8^ CAR+ T cells	39 (95%) received CTX130 objective response rate (ORR) 18/39 patients (46.2%) complete response (CR) 6/39 (19.4%) partial response (PR): 10/39 (32.3%) median follow-up: 7.4 months	CRS G1–2, neurotoxicity (10%) mild (G1–2) grade 3–4 AEs: neutropenia (36%), 21 deaths: 16 from disease progression and 5 unrelated to CTX130
Reef et al. [126]	2024	6	F:1 M:5	CCR4.CD30	Flu 30 mg/m^2^/d, Benda 70 mg/m^2^/d −5, −4, and −3 d (+/− 1 d) or Cy 500 mg/m^2^/d for 3 d before CAR T infusion (d0), 3 + 3 escalation: DL1/3/5—CCR4.CD30 CAR-T (2 × 10^7^, 5 × 10^7^, 1 × 10^8^/m^2^); DL2/4/6—same dose + CD30 CAR-T (1 × 10^8^/m^2^)	median skin tumor reduction of 42.2% 50% of patients achieved stable disease none progressed, but all required further therapy median overall survival was 23.9 months median follow-up 26.0 months	No CRS or ICANS, grade 3–4 AEs: hematological, G3 diverticulitis (*n* = 1), G3 neutropenia with infection (*n* = 1).

Abbreviations: AE—adverse event, Benda—bendamustine, CAR T—chimeric antigen receptor T cell, CCR4—C-C chemokine receptor 4, CCR4-CAR T—C-C chemokine receptor 4 chimeric antigen receptor T cell, CD30—cluster of differentiation 30, CD4+—CD4-positive T lymphocyte (T helper cell), CD70—cluster of differentiation 70, CRS—cytokine release syndrome, CTCL—cutaneous T-cell lymphoma, Cy—cyclophosphamide, Flu—fludarabine, ICANS—immune effector cell-associated neurotoxicity syndrome, IV—intravenously,, NR – not reported, TAG-72—tumor-associated glycoprotein 72, Th17—T helper 17 cell, Th2—T helper 2 cell, Treg—regulatory T cell.

**Table 5:** Roadblocks and challenges in CAR T-cell therapy.

Challenges	Solutions/Future Directions	References
Autoimmune disorders (SLE and SSc) SLE		
Limited durability of immune reset due to persistence of long-lived plasma cells and tissue-resident immune niches Risk of re-emergence of autoreactive clones despite initial B-cell depletion Impaired immune reconstitution (infection, hypogammaglobulinemia) Disease heterogeneity and unclear responders Uncertain long-term immune effects	CD19-mediated immune reset; dual-target CARs (CD19/BCMA) Controlled B-cell reconstitution BAFF-targeted combinations Autoreactive clone-specific approaches (CAARs) Next-generation CAR designs (logic-gated, safety switches) In vivo CAR engineering (mRNA/LNP, viral vectors) Biomarker-driven patient selection	[43,47,92,160,161,162,164,165,180,181,182,240,241,242]
SSc		
Limited reversibility of established fibrosis Unclear antifibrotic vs. immunomodulatory effects Persistence of plasma cells and fibrotic memory circuits Strong dependence on disease stage Heterogeneity and lack of predictive biomarkers	Early intervention before irreversible fibrosis Dual-target strategies (CD19/BCMA) In vivo CAR platforms; longitudinal tissue-level assessment of antifibrotic effects Targeting immune–stromal interactions Biomarker-driven patient stratification Integration with antifibrotic approaches Tissue-level monitoring and long-term trials	[51,52,93,94,95,96,97]
Melanoma		
Immunosuppressive tumor microenvironment (TGF-β, PD-L1, adenosine) → T-cell exhaustion Physical barriers limiting infiltration (dense ECM, CAFs, abnormal vasculature) Tumor heterogeneity and antigen loss → immune escape On-target/off-tumor toxicity due to shared antigen expression Intrinsic resistance to apoptosis (Bcl-2, IAPs, ↓ death receptors)	Armored CAR T-cells (IL-12, IL-15, TGF-β resistance) ECM remodeling (e.g., HPSE); chemokine-axis modulation Multi-antigen CARs; safety engineering (affinity tuning, suicide switches) Combination therapies (checkpoint inhibitors, oncolytic viruses, epigenetic drugs) Universal and in vivo CAR platforms Multimodal TME reprogramming	[156,183,185,186,187,188,189,190,191,192,193,194,195,196,197,198,199,200,201,202,203,204,205,206,207,208,209,210,211,212,213,214,215]
Cutaneous T-cell lymphoma		
High antigen overlap between malignant and normal T cells → severe off-tumor toxicity Tumor heterogeneity and lack of universal tumor-specific antigen Risk of global T-cell depletion → immunosuppression and infection Limited antigen exclusivity and clonal variability	Multi-target CARs and logic-gated systems (AND/OR/NOT) to improve specificity Targeting more selective antigens (KIR3DL2, TRBC1) TRBC1-directed CAR T-cells enabling clonal targeting with partial T-cell preservation Exploration of alternative targets (CCR4, CD70, TAG-72) with improved selectivity	[42,216,217,218,219,220,221,222,223,224]

## Data Availability

No new data were created or analyzed in this study. Data sharing is not applicable to this article.
